# Hemorrhage contributes to chronic adverse remodeling in acute myocardial infarction: Insights from a novel preclinical model

**DOI:** 10.1186/1532-429X-17-S1-P111

**Published:** 2015-02-03

**Authors:** Nilesh R Ghugre, Nancy Shie, Xiuling Qi, Mihaela Pop, Jennifer Barry, Bradley H Strauss, Graham A Wright

**Affiliations:** Physical Sciences Platform, Sunnybrook Research Institute, Toronto, ON Canada; Department of Medical Biophysics, University of Toronto, Toronto, ON Canada; McGill University, Montreal, QC Canada; Schulich Heart Program, Sunnybrook Health Sciences Centre, Toronto, ON Canada

## Background

Hemorrhage in association with microvascular obstruction (MVO) is a new independent predictor of adverse remodeling following acute myocardial infarction (AMI), occurring in ~35% of patients presenting with STEMI [1, 2]. However, it remains unsettled whether hemorrhage is simply a marker of severity or directly contributes to the ongoing remodeling process. The aim of our study was to to probe the downstream consequences of hemorrhage in chronic remodeling following AMI by employing a novel minimally-invasive model of myocardial hemorrhage in an experimental setting.

## Methods

Myocardial hemorrhage was induced in a porcine model of AMI by direct intracoronary injection of collagenase (col) [3]. Animals (N=12) were divided into three groups based on the type ischemia-reperfusion injury inflicted in the left anterior descending artery (LAD) - Group 1 (N=3) 45 min occlusion with saline; Group 2 (N=5): 8 min ischemia with col; and Group 3 (N=4): 45 min occlusion with collagenase. Imaging was performed on a 3T MRI scanner (MR 750, GE Healthcare) serially at baseline (healthy state) and day 1, week 1 and week 4 post-AMI. Cardiac function was assessed with cine SSFP; edema with T2 mapping; hemorrhage with T2* mapping; MVO and infarction with early and late gadolinium enhancement (EGE, LGE) respectively.

## Results

**Figure 1:**
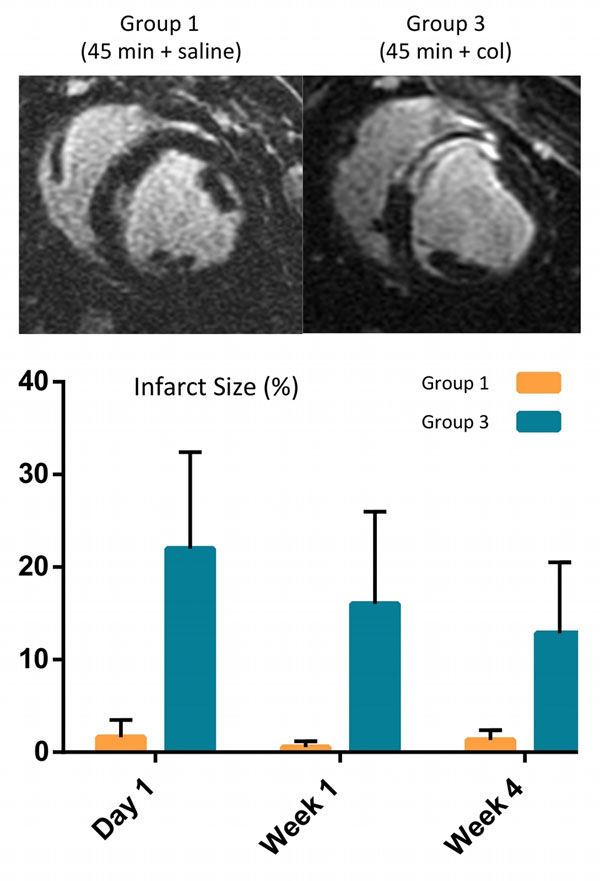
Representative late gadolinium enhancement (LGE) short-axis images from animals in Groups 1 and 3 along with the infarct size evolution post-AMI.

**Figure 2:**
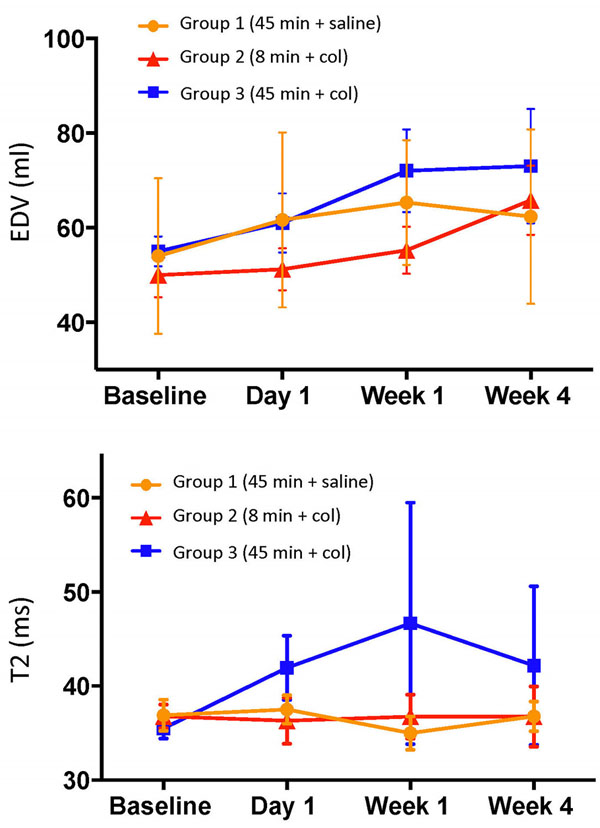
Time evolution of end diastolic volume (EDV) and T2 relaxation time in the infarct zone over 4 weeks in each of the three groups post-AMI.

At day 1, low T2* values in the infarct region confirmed the presence of myocardial hemorrhage in the collagenase groups 2 and 3 (16 ms vs. 33 ms at baseline, p<0.001) whereas group 1 was non-hemorrhagic. Infarct size was significantly greater in group 3 compared to group 1 at all time points (Fig. 1, p<0.0001); group 2 did not show any infarction on LGE images. MVO was present only in group 3 (1 of 4 animals). End diastolic volume (EDV) was significantly elevated at week 4 compared to baseline in both the hemorrhagic groups 2 and 3 (p<0.001) whereas in group 1, it was unaffected (Fig. 2); ejection fraction (EF) was not significantly different between the groups possibly due to compensatory mechanisms. Infarct zone edema as indicated by elevated T2, was greatest in group 3 compared to the other two groups at week 1 (p<0.05) and persisted at week 4 (p=NS). Trends (p=NS) at day 1 and week 4 indicated that wall motion was more depressed in the infarct zone of group 3 (1.2 mm, 1.9 mm) compared to groups 1 (2.8 mm, 2 mm) and 2 (2.7 mm, 3.5 mm), respectively.

## Conclusions

Our study demonstrates that the introduction of myocardial hemorrhage at reperfusion results in greater myocardial and microvascular damage, upregulated inflammation, and chronic adverse remodeling (increased preload stress) beyond the effects of the initial ischemic insult. Thus, hemorrhage actively contributes to the tissue remodeling processes during infarct healing. A mechanistic view of the consequences of hemorrhage post-AMI will potentially lead to better management and care of the high-risk STEMI patient population.

## Funding

We acknowledge funding support from the Heart & Stroke Foundation of Canada, GIA award #000334, and the D+H Sunnybrook Research Institute (SRI) Summer Student Program.
